# Genome-wide identification of the *B3* transcription factor family in pepper (*Capsicum annuum*) and expression patterns during fruit ripening

**DOI:** 10.1038/s41598-023-51080-6

**Published:** 2024-01-26

**Authors:** Tao Wang, Cha Long, Meixia Chang, Yuan Wu, Shixian Su, Jingjiang Wei, Suyan Jiang, Xiujun Wang, Jianwen He, Dan Xing, Yangbo He, Yaoqi Ran, Wei Li

**Affiliations:** 1 College of Agriculture, Guizhou University, Guiyang, 550025 China; 2 Vegetable Research Institute, Guizhou University, Guiyang, 550025 China; 3 Engineering Research Center for Protected Vegetable Crops in Higher Learning Institutions of Guizhou Province, Guiyang, 550025 China; 4 College of Brewing and Food Engineering, Guizhou University, Guiyang, 550025 China; 5 Pepper Research Institute of Guizhou Province, Guiyang, 550006 China; 6 Agriculture Development and Research Institute of Guizhou Province, Guiyang, 550006 China

**Keywords:** Developmental biology, Molecular biology, Plant sciences

## Abstract

In plants, *B3* transcription factors play important roles in a variety of aspects of their growth and development. While the *B3* transcription factor has been extensively identified and studied in numerous species, there is limited knowledge regarding its *B3* superfamily in pepper. Through the utilization of genome-wide sequence analysis, we identified a total of 106 *B3* genes from pepper (*Capsicum annuum*), they are categorized into four subfamilies: RAV, ARF, LAV, and REM. Chromosome distribution, genetic structure, motif, and cis-acting element of the pepper *B3* protein were analyzed. Conserved gene structure and motifs outside the *B3* domain provided strong evidence for phylogenetic relationships, allowing potential functions to be deduced by comparison with homologous genes from Arabidopsis. According to the high-throughput transcriptome sequencing analysis, expression patterns differ during different phases of fruit development in the majority of the 106 *B3* pepper genes. By using qRT-PCR analysis, similar expression patterns in fruits from various time periods were discovered. In addition, further analysis of the *CaRAV4* gene showed that its expression level decreased with fruit ripening and located in the nucleus. *B3* transcription factors have been genome-wide characterized in a variety of crops, but the present study is the first genome-wide analysis of the *B3* superfamily in pepper. More importantly, although *B3* transcription factors play key regulatory roles in fruit development, it is uncertain whether *B3* transcription factors are involved in the regulation of the fruit development and ripening process in pepper and their specific regulatory mechanisms because the molecular mechanisms of the process have not been fully explained. The results of the study provide a foundation and new insights into the potential regulatory functions and molecular mechanisms of *B3* genes in the development and ripening process of pepper fruits, and provide a solid theoretical foundation for the enhancement of the quality of peppers and their selection and breeding of high-yield varieties.

## Introduction

Members of the *B3* transcription factors are the superfamily specific to plants, and they all have at least one region known as the *B3*-binding structural domain^1,2^. The third basic region of VIVIPAROUS 1 (VP1) in maize is where the structural domain is first identified^3^. Around 110 amino acid residues of recognizable DNA make up the conserved *B3* structure region, which has two short α-helices and seven β-barrels^1,2^. As far as we know, the *B3* superfamily is composed of four subfamilies, including RELATED TO ABI3/VP1 (RAV), AUXIN RESPONSE FACTOR (ARF), LEAFY COTYLEDON2-ABSCISIC ACID INSENSITIVE3-VAL (LAV), and REPRODUCTIVE MERISTEM (REM), analyzed genetic structure and systemic development^1^. The *B3* superfamily has been found in numerous species including mustard, rice, grapes, cucumbers, maize, bryophytes, pineapple, oak, cocoa, soybeans, *Brassica rapa*, *Arabidopsis thaliana*, tobacco, hazelnuts, eagle beans, citrus, spinach, and red algae^4–12^. However, it has not been detected in significant vegetable crops such as peppers. Members of the *B3* superfamily serve critical regulatory activities that are required for plant growth and stress response. RAV subfamily proteins have a *B3* domain at the end that identifies the CACCTG motif. Additionally, certain RAV subfamily members include an AP2/EREBP domain that binds to the CAACA sequence at the beginning^13^. Members of the RAV subfamily control the formation of lateral roots, the timing of flowering, and seed growth. Additionally, they assist in signal transmission, biotic and abiotic stress response, and leaf senescence promotion^14–21^. LAV subfamily members each have a single *B3*-binding structural domain that recognizes the Sph / RY element of the CATGCA sequence. Each of them also possesses a motif called ethylene response element binding factor-associated amphipathic repressor (EAR), which inhibits the expression of genes linked to seed maturation by acting as a transcriptional repressor. Additionally, certain ones include the zf-CW domains, which change the dynamics and structure of chromosomes, interact with histone methylation, and regulate gene expression. The LAV subfamily mainly regulates seed development, induction of somatic embryogenesis, embryonic development, and adversity stresses^6,22–28^. The REM subfamily typically consists of several *B3* binding domains, although there hasn't been much research done on the unique DNA-binding capacity of the REM subfamily's *B3* domain. The genes *REM1*, *VRN1*, and *VDD* play a role in various processes including cell division, formation of floral tissues, development of embryos, and pollen development^29–33^. Furthermore, while the DNA binding specificity of *ARF*, *RAV*, *LAV*, and *REM* has been studied, few research have thoroughly examined the structure, expression, and function of the *B3* superfamily in plants.

The ARF subfamily of the *B3* gene may be essential for fruit growth and maturity. The ARF subfamily is a part of the auxin-responsive factors and contains a *B3*-binding domain at the beginning, which identifies the TGTCTC element (indole-3-acetic acid -responsive) in gene promoter that respond to growth hormone. Furthermore, it has a C-terminal AUX/IAA-interacting domain that promotes interactions between ARFs and AUX/IAA inhibitors, as well as an intermediate region to determine whether ARF activates or represses target genes^34,35^. The ARF subfamily members play a role in controlling various developmental processes influenced by auxin, including the establishment of adventitious roots, the growth of flowers and fruits, the production of seeds^36–40^. A few studies have been carried out on the ARF subfamily in fruit development and maturation, as mentioned in certain reports. For instance, in *Arabidopsis thaliana*, the inhibition of carpel development into fruits is observed due to *ARF8*, and mutations in *ARF8* lead to the formation of seedless fruits without the need for pollination and fertilization^41^. Additionally, the closure of germ in infertile flowers is hindered in *ARF8*/*ARF6* double mutants, and flower maturity is completely blocked in *ARF6*/*ARF8* mutants^42^. Recent studies have revealed that *ARF* genes affect tomato fruit set, development, ripening, and quality^43–47^. Little is known about how ARF members govern fruit development in various plants since the regulatory mechanism of fruit development is frequently species-specific.

Pepper (*Capsicum annuum*) is a plant in the Solanaceae that is widely grown for both vegetables and spices^48^. Due to its considerable diversity in color and form^49^, it offers a commendable model for investigating the growth of non-climacteric fruits. Fruit development and ripening are physiological processes specific to plants, mainly involving expansion, sweetening, and increased pigmentation^50,51^. Significant physiological, biochemical, morphological, and molecular changes exist at this stage, with phytohormones and transcription factors being determinants of fruit growth, development, and ripening^52,53^. The transcription factors MADS-box, MYB (v-myb avian myeloblastosis viral oncogene homolog), NAC (NAM/ATAF1/CUC2), and SPL (squamosa promoter-binding protein-like) all contribute to the development of fruits^54–57^. Despite the *B3* transcription factor's significance in regulating fruit growth, an exhaustive study of the *B3* superfamily in pepper has not yet been carried out. More importantly, since the molecular mechanisms of pepper fruit development and maturity processes have not been fully explained, it is unclear whether the *B3*-transcript factor regulates pepper fruit development and maturity.

We examined the genome-wide analysis of the *B3* superfamily in pepper for this study. The pepper genome contains 106 *B3* genes, whose chromosomal locations, gene structures, promoter cis-acting elements, subcellular locations, and phylogenetic relationships have been examined. We also studied at the conserved motifs and biochemical characterization of *CaB3* proteins. In addition, we used high-throughput transcriptome analysis to discover many *CaB3* genes linked to pepper fruit maturity. Quantitative Real-time PCR (qRT-PCR), a technique that generates numerical data, was used to confirm the expression patterns of the selected genes. The main goal of this work was to increase knowledge of the possible role of the *B3* superfamily gene in the maturation and development of pepper fruits.

## Results

### Identification of *B3* genes in the pepper genome

The pepper (*C. annuum*) genome contained a total of 106 genes belonging to the *B3* superfamily. The putative pepper *B3* proteins exhibited significant variation in their amino acid lengths, with the 106 *B3* superfamily genes encoding proteins ranging from 90 to 1186 amino acids. Additionally, these proteins range from 4.47 to 10.49 in isoelectric point and 10.34–133.98 KDa in molecular weight. Based on their homologous sequences in *Arabidopsis thaliana*, the four subfamilies RAV, ARF, LAV, and REM are used to group the 106 members of the pepper *B3* superfamily^1,6^ (Supplementary Table S1). In pepper, *REM* has been found to be the largest *B3* subfamily, with a total of 70 *CaREM* genes identified. *ARF* is the second largest subfamily, with 23 *CaARF* genes identified; The *LAV* and *RAV* subfamilies are much smaller, with eight *CaLAV* and five *CaRAV* genes respectively identified in pepper, accounting for 66%, 21.7%, 7.6%, and 4.7% of the total identified B3 family genes, respectively.

### Phylogenetic analysis of the pepper *B3* genes

**Figure 1:**
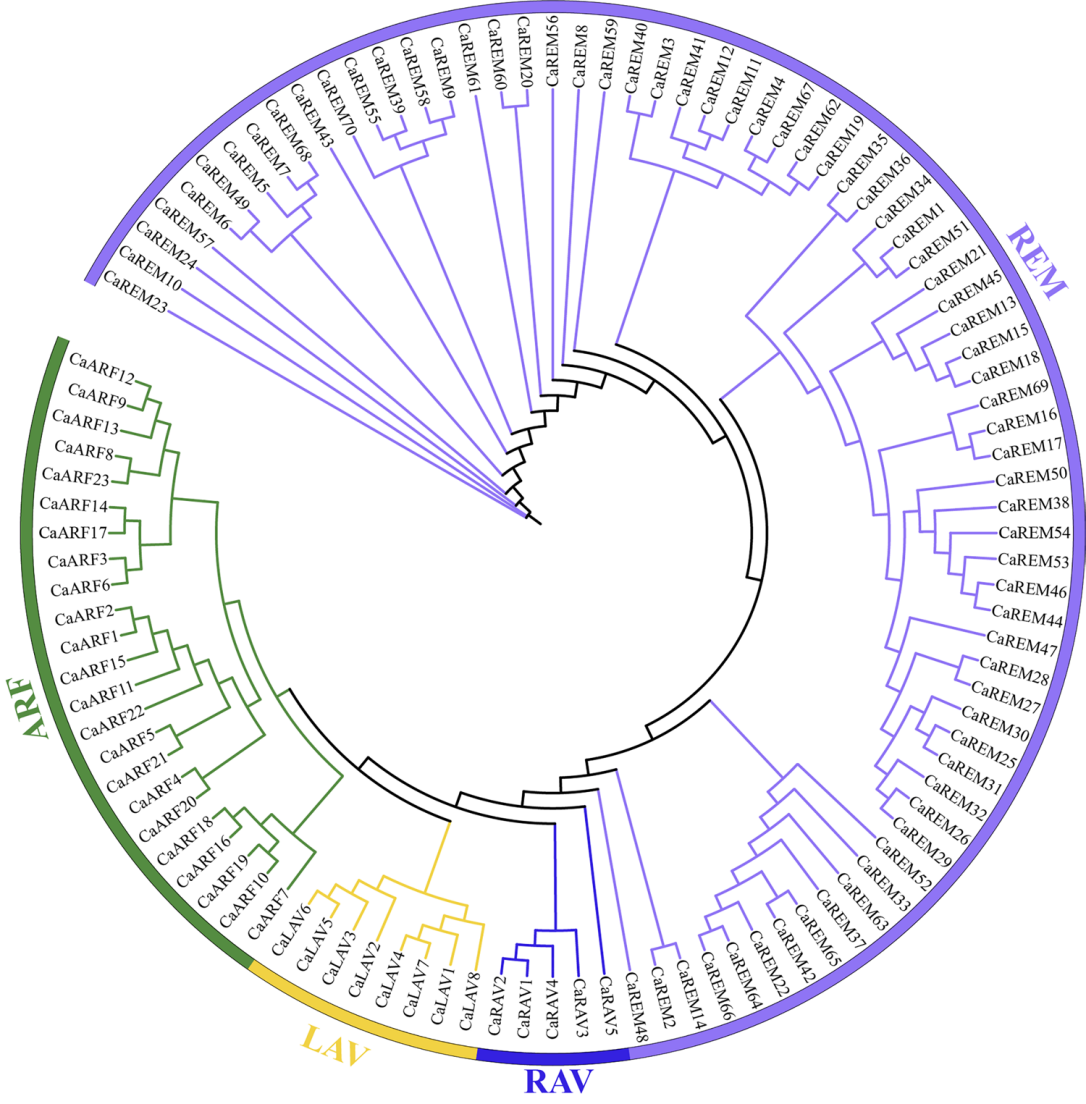
The phylogenetic tree of pepper *B3s*, with distinct subfamilies (REM, RAV, LAV, ARF) represented by various color regions.

**Figure 2:**
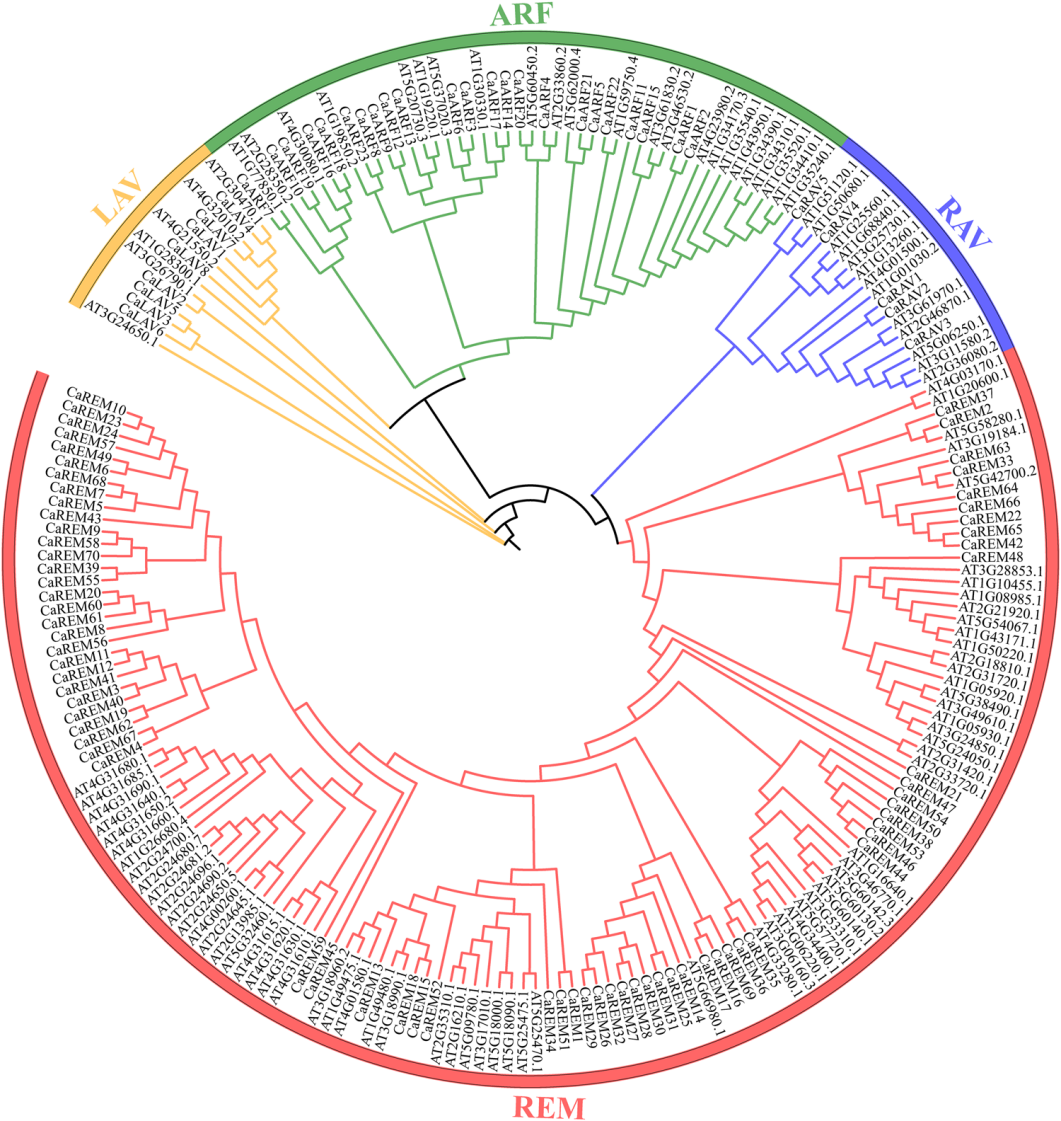
The phylogenetic tree displays the *B3s* from pepper (Ca)and Arabidopsis (At), with distinct color areas representing various subfamilies (REM, RAV, ARF, LAV).

A phylogenetic tree was generated for the *B3* genes in pepper in order to gain insight into the evolutionary relationships within the *B3* superfamily (Fig. 1). The findings indicated that the pepper *B3* superfamily genes were categorized into four primary lineages, exhibiting notable variations in the member count within each cluster. Excluding *CaRAV5* and 10 REM members (*CaREM23, CaREM10, CaREM24, CaREM57, CaREM43, CaREM61, CaREM56, CaREM8, CaREM59, and CaREM48*), the *B3* proteins of the same subfamily formed a cluster in the same branch during the analysis. Furthermore, 23 *ARF* subfamily genes were mostly distributed in nine sister pairs (*CaARF*12/9,*CaARF*8/23,*CaARF*14/17,*CaARF*3/6,*CaARF*2/1,*CaARF*5/21,*CaARF*4/20,*CaARF*18/16,*CaARF*19/10) with the remaining five *CaARFs* not matching from *CaARF12* to *CaARF 7* (from left to right). In the REM subfamily, there were only 20 paralogous pairs for 70 genes, and the remaining 30 genes were mismatched, whereas there were one (*CaRAV*1/2) and two paralogous pairs(*CaLAV*5/6, *CaLAV*4/7) for each of the five *RAV* subfamily genesand eight subfamily genes, respectively. The direct homology of the *CaB3* genes in various subfamilies is illustrated by this. Upon comparing the evolutionary trends of *B3* proteins in pepper and *Arabidopsis thaliana* (Fig. 2, Supplementary Table S2), RAV, ARF, and REM subfamilies were shown to cluster together with members of their respective subfamilies on a single branch. Notably, all the major branches and sub-branches included interspecific members. Nevertheless, REM subfamily members exhibit distinct distribution patterns across various branches of the phylogenetic tree, with the majority consisting of intraspecific members. These results illustrate that one of the fundamental features of the REM subfamily is its relative diversity^6^.

### Chromosomal distribution of the pepper *B3* genes

**Figure 3:**
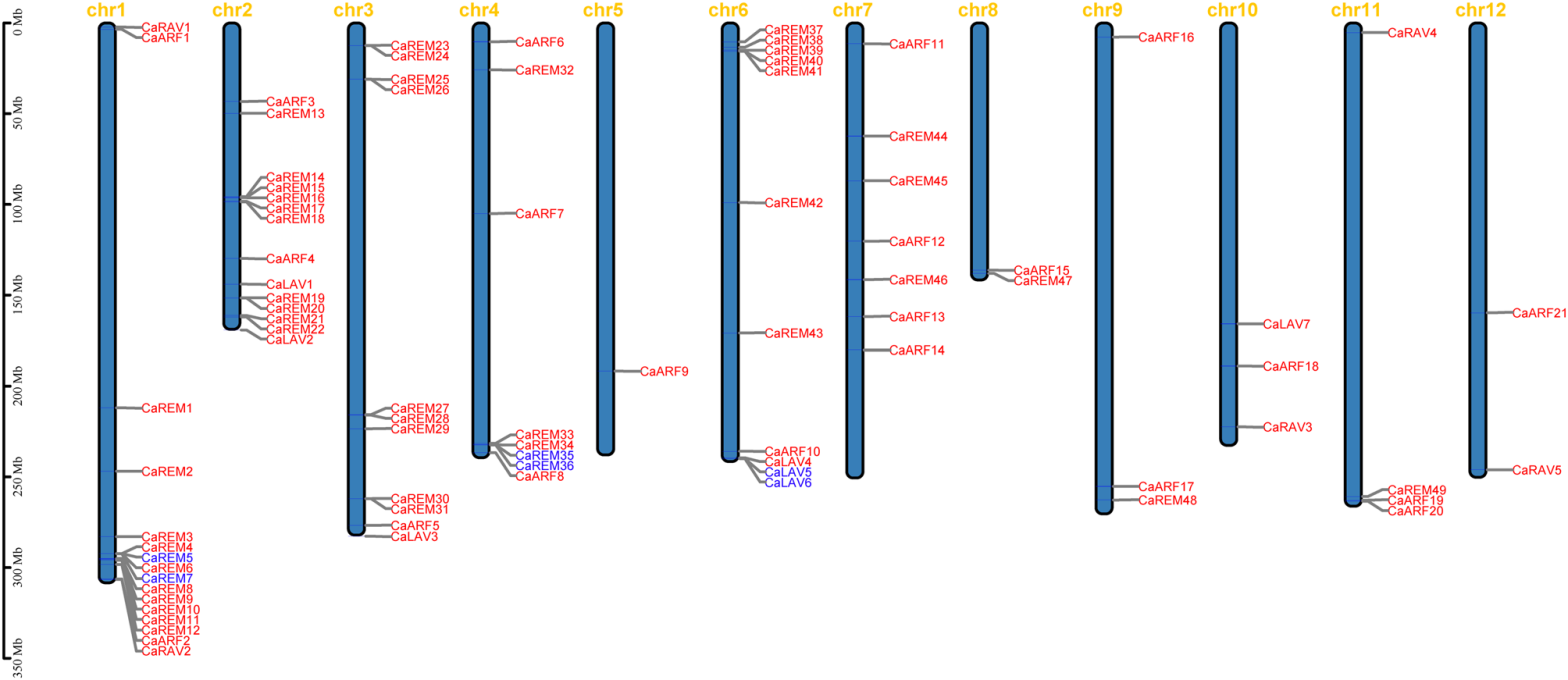
The 12 pepper chromosomes contain *B3* genes, whose location, distribution, and tandem duplication. Each chromosome is labeled with its respective chromosome ID at the top. Blue font indicates tandem duplicated gene pairs. The scale is measured in megabases (Mb).

We performed a mapping study to find the distribution of the pepper *B3* genes across chromosomes in order to better understand the chromosome location of the pepper *B3* superfamily (Fig. 3). In addition to the 24 *CaB3s* found on various scaffolds (Supplementary Table S1), the rest of the *CaB3s* were unevenly distributed among the 12 linkage groups (LG) of pepper. The density of *CaB3* genes per chromosome was variable, with only one gene (0.9%) on chromosome 5 (*CaARF9*) and as many as 16 genes on chromosome 1 (15.1%). At the extremities of the chromosomes, *CaB3* genes showed relatively high concentrations, with the lower end of chromosome 1 having the highest concentration. It is important to note that there is no direct relation between the number of *CaB3* genes and chromosomal length. For instance, chromosome 2, despite being shorter, contains 14 *CaB3* genes, while chromosome 5, which is longer, only has one *CaB3* gene.

### Collinearity and duplication analysis of the *B3* genes in pepper

**Figure 4:**
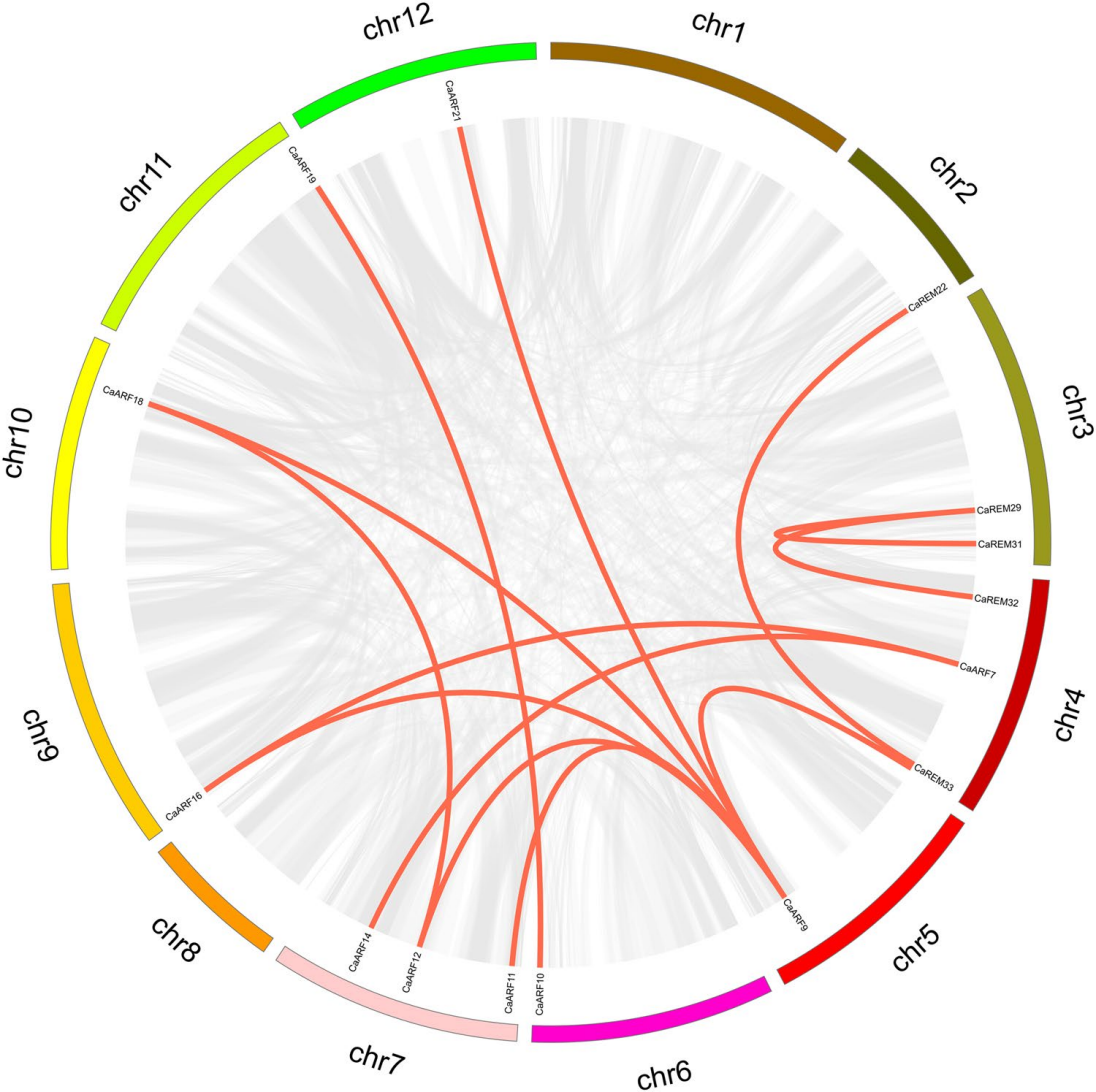
A Collinearity of *CaB3* genes in pepper. Duplicated *CaB3* pairs are indicated by the red lines.

In order to enhance our comprehension of the expansion patterns and evolutionary connections within the pepper *B3* superfamily, we conducted analyses on duplication events and collinearity. The findings indicated that out of all the pepper *B3* genes identified, there were a combined total of four instances of tandem duplication on chromosomes 1, 4, 6, and scaffold757 (Supplementary Tables S1, S2 and Fig. 3). Additionally, 13 occurrences of segmental duplication were observed on 10 chromosomes, excluding chromosomes 1 and 8 (Fig. 4). The expansion of the pepper *B3* superfamily may be influenced by segmentation and the replication process occurring in tandem. Calculating the ratios of Ka (non-synchronous substitution rate) and Ks (symmetric substitution ratio) is beneficial for evolutionary study. The results showed that the Ka/Ks (non-synonymous/synonymous substitution rate) ratios in the pepper *B3* superfamily ranged from 0.15 to 0.89, all of which were below 1. These ratios were caused by the duplication of gene pairs.

**Figure 5:**
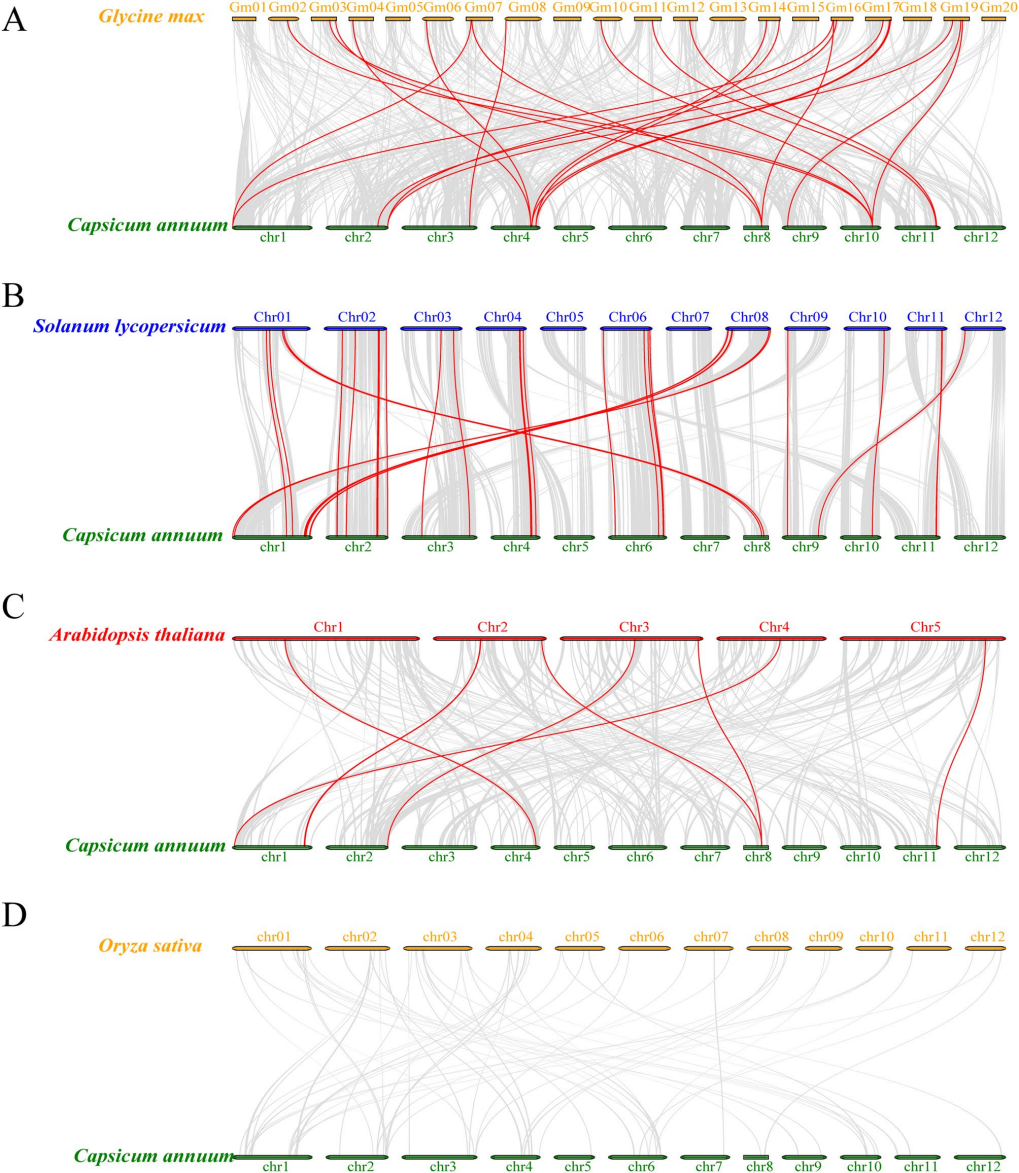
(**A**). Collinearity Analysis the correlation between pepper and soybean. (**B**). Analysis of collinearity between pepper and tomato plants. (**C**). Collinearity analysis was conducted between pepper and Arabidopsis plants. (**D**). Analysis of collinearity between pepper and rice. Duplicated *CaB3* pairs are shown as red lines, while segmental duplications are shown as gray lines.

Collinearity studies were performed on the dicots (soybean, tomato, and *A. thaliana*), as well as the monocots (rice), so as to more thoroughly examine the evolutionary process of the pepper *B3* superfamily (Fig. 5). According to the findings, *AtB3*, *SlB3*, and *GmB3* exhibited collinearity with 8, 44, and 13 *CaB3* genes, respectively, while no collinearity was observed with *OsB3*.The above crops formed 9, 44, and 23 pairs of orthologous genes (Supplementary Table S4). These differences may have been influenced by variations in the timing of evolutionary divergence of the *B3* gene among species.

### Structure and protein motif analyses of the pepper *B3* genes

**Figure 6:**
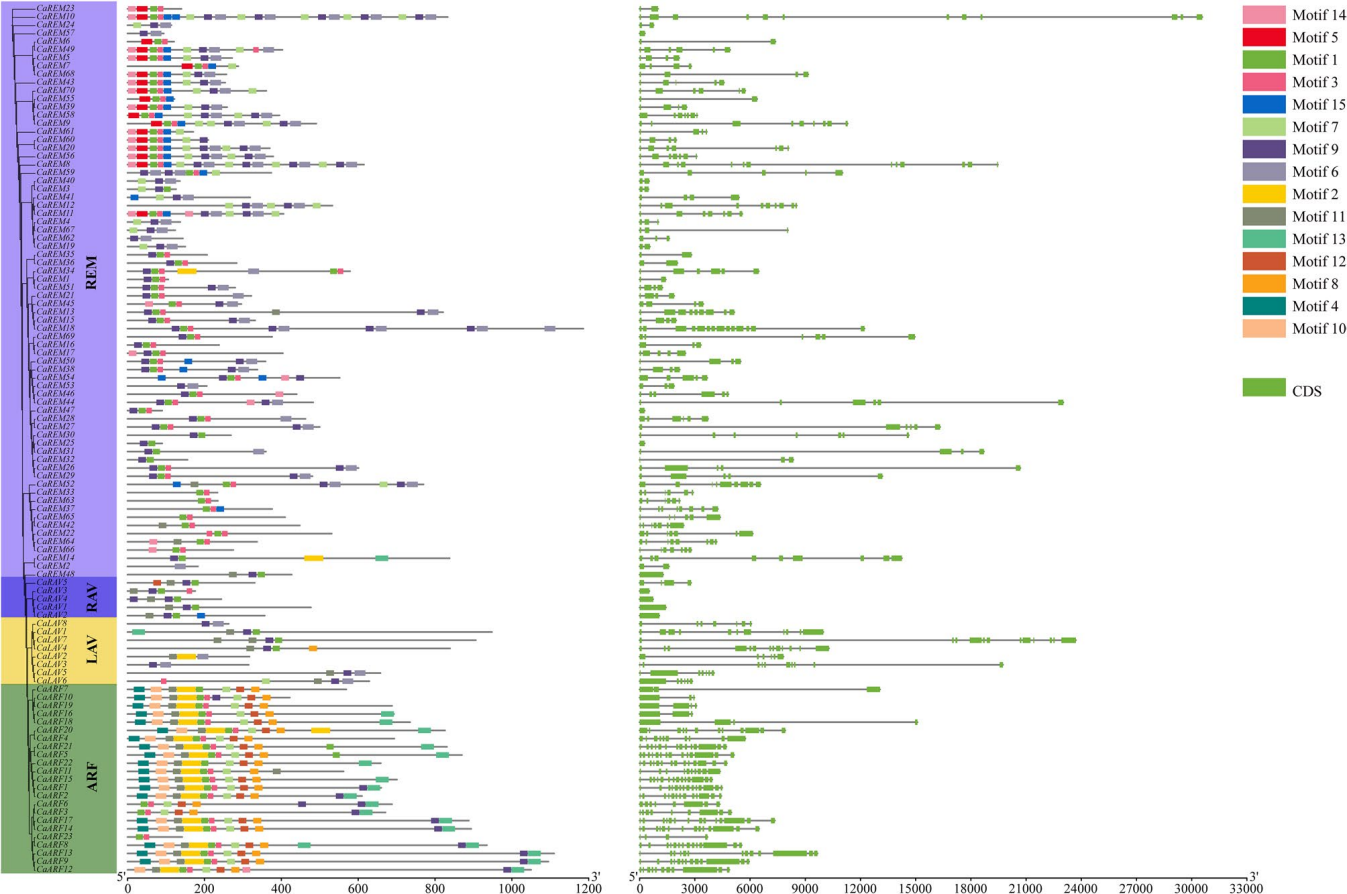
The exon–intron structure and motif organization of *CaB3s.* (**A**). Phylogenetic relationships of *CaB3s*. (**B**). Motif identification of *CaB3* proteins using MEME. (**C**). The intron and exon structures of *B3* genes. Exons are shown by the green boxes, while introns are symbolized by the lines through the boxes.

The emergence of structural polymorphism in genes during the evolution of the superfamily is common and can assist the co-selection of genes for additional functions in response to environmental changes^58^. Out of the 106 *CaB3s* that are categorized into four subfamilies, the majority of *REM* subfamily genes consist of 4–14 exons, while ARF subfamily genes possess more than three exons. *RAV* subfamilies, on the other hand, exclusively contain either one or four exons, whereas *LAV* subfamilies exhibit a range of 6–13 exons (Fig. 6). Within the *REM* subfamily, adjacent genes may exhibit variations in the number of exons within their respective phylogenetic trees. In addition, the examination of motifs revealed 15 distinct sequences within the *CaB3* protein sequence, and the findings indicated that all *CaB3* genes possess a designated B3 domain motif. The same family had similar patterns, which differed greatly among the different subfamilies. For instance, the *RAV* genes possess motif6 and motif9, while the *ARF* genes possess motif1 and motif3. On the other hand, motif5 is exclusively present in the *REM* genes, and the *ARF* family exclusively contains motif4, motif12, and motif10 (Fig. 6, Supplementary Figure S1). The various patterns of the subfamily genes within the population indicate that the *CaB3* superfamily has multiple functions.

### Cis-acting element analysis of the pepper *B3* genes

**Figure 7:**
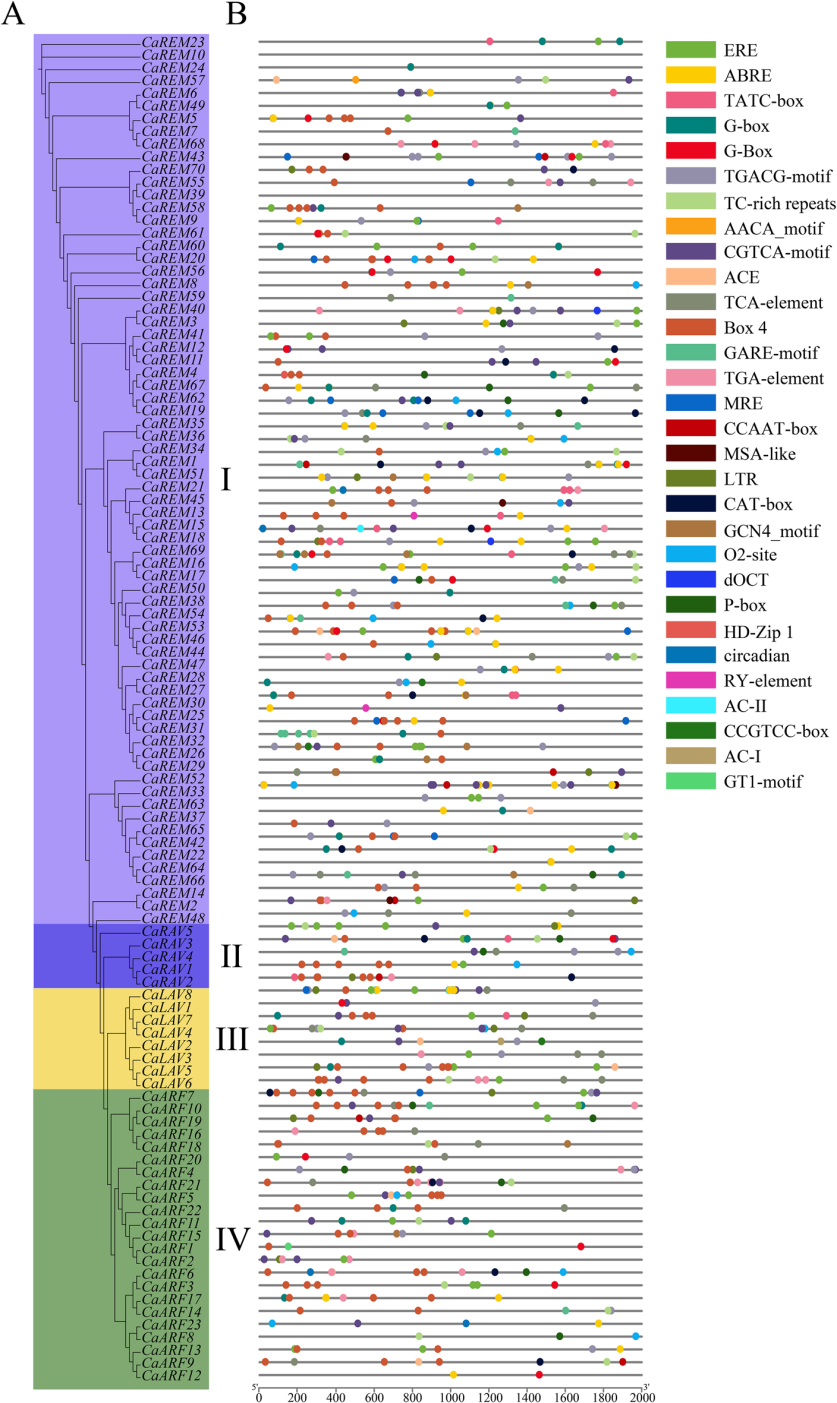
Prediction of cis- acting elements in the *B3* genes of pepper. (**A**). Phylogenetic relationships of *CaB3s*. (**B**). Prediction of cis-elements in *CaB3* promoters. The *CaB3* gene is colored to signify various cis-acting elements and their corresponding positions. The promoter is 2 kb long. The promoter is 2 kb in length.

The contact of the RNA polymerase with the promoter is a critical event in the initial phase of transcription, which is a critical step of gene expression. RNA polymerase binding affinity and gene expression level are influenced by the promoter's structure^59^. To further comprehend *CaB3s*' possible regulatory functions in plant development as well as growth, an analysis was conducted on a promoter sequence that is 2000 base pairs long preceding ATG. An overall number of 799 cis-acting elements were observed in the promoters of 106 *CaB3s* (Fig. 7, Supplementary Table S5). These components are divided into four categories: hormonal reactions, photoreactions, coercive responses, and growth and developmental regulation. Within the REM subfamilies, the most extensive category of cis-acting elements is comprised of hormone-responsive ones, making up 49.8% of all cis-acting elements. It responded more to abscisic acid, methyl jasmonate, and ethylene than the other components. Hormone-sensitive cis-acting elements comprised the majority (46.4%) of cis-acting elements in the ARF subfamily, exhibiting a higher response to methyl jasmonate, abscisic acid, and ethylene compared to other components. Hormone-sensitive cis-regulatory elements in the LAV subfamily constituted the majority, making up 52.6% of all cis-regulatory elements. Compared to other compounds, these elements responded more strongly to salicylic acid, ethylene, and methyl jasmonate. Hormone-responsive cis-acting elements made up 46.7% of all cis-acting elements in the RAV subfamily, making them the most prevalent kind. These elements exhibited a stronger response to methyl jasmonate, ethylene, and gibberellin compared to other components. Furthermore, a total of 270 (33.8%) cis-regulatory elements that are sensitive to light were discovered across all *CaB3* genes. These elements consist of light-responsive components as well as GT binding sites that are linked to the light response. In addition, a total of 48 (6.0%) elements that respond to stress were found, which encompass stress-responsive elements, defense elements, and low-temperature responsive elements. Additionally, 68 (8.5%) elements linked to growth and developmental responses were found, such as elements regulating meristematic tissue development, transcription factors binding to CCAAT-box, elements related to fenestrated tissue cell differentiation, elements regulating circadian rhythm, elements regulating cell cycle, elements controlling zein metabolism, and elements specific to seed.

### Expression patterns of *B3* genes during pepper fruit ripening

In this study, we gathered pepper fruits at three distinct stages of development: mature green fruit (MG, 40 DPA), breaker fruit (BR, partially red fruits, 50 DPA), and red ripe fruit (RR, completely red fruits, 60 DPA). In order to find the expression of the *B3* gene during fruit ripening in pepper, we next carried out high-throughput transcriptome sequencing analysis to look at the pattern of gene expression in the process of growth and ripening of the peppers. In the three stages of fruit development, it was found that 93 *CaB3* genes had FPKM values greater than zero, while the expression of 13 *CaB3* genes was undetectable throughout the three developmental phases (Supplementary Table S6) illustrates distinct expression patterns observed in the majority of pepper *B3* genes across the three developmental stages. Within the LAV subfamily, the expression levels of *CaLAV1/4/7/8* were higher throughout the three stages of fruit development, whereas *CaLAV2/4/6* exhibited lower expression. The transcription level of *CaLAV3* gene is zero. Genes related to the RAV subfamily have low expression levels in general. However, *CaRAV1* exhibited the highest transcriptional abundance during red maturity, while *CaRAV4* showed higher levels during green and broken maturity. The three phases of fruit development have shown high levels of expression for the ARF subfamily *CaARF14/5/6*, whereas the expression of *CaARF2/10/18* has been observed to be very low in these three phases. The transcript abundance of REM subfamily was comparatively lower than other subfamilies, particularly *CaREM1/3/6/7/19/25/32/36/40/44/46/57*, which showed no expression throughout all three fruit developmental periods. However, *CaREM21/22/49/53* had higher transcript abundance, suggesting the varied expression patterns within the REM subfamily. During the three developmental periods, there were duplicated gene pairs that exhibited diverse expression profiles. For instance, *CaARF14* displayed high expression levels (FPKM ranging from 68.83 to 79.01), whereas its segmental duplicated gene, *CaARF7*, had low expression levels (FPKM between 2.63 and 3.73). These results suggest that functional divergence between the duplicated genes may have occurred throughout the process of evolution.

In order to further verify the dynamic expression pattern of *CaB3* genes in different developmental stages of fruit, we selected five up-regulated genes (*CaRAV1*, *CaREM61*, *CaREM62*, *CaARF8*, *CaARF10*), and four down-regulated genes(*CaRAV4*, *CaREM17*, *CaREM48*, *CaARF1*) on the basis of their relatively high multiplicity of differences in transcript abundance at different stages (|log2FC|≥ 1, *P* ≤ 0.05), and the transcript levels of nine *CaB3* genes were investigated by qRT-PCR to examine the relative expression levels of *CaB3* genes at three developmental stages of pepper fruits. The transcriptome results agreed with the expression patterns of these genes during the three phases of development. During pepper fruit maturation (Fig. 9), the findings indicated significant expression of four *CaB3* genes, namely *CaRAV1*, *CaREM61*, *CaREM62*, and *CaARF8*.Furthermore, the levels of *CaREM62*, *CaREM61*, *CaARF8*, and *CaRAV1* were markedly increased in MG-BR-RR during the RR period, with increases of 2.18 times, 2.60 times, 3.19 times, and 9.17 times, in contrast to the BR period. Conversely, the expression of CaARF10 was initially decreased by 1.16 times in Mg-BR and subsequently increased by 2.63 times in BR-RR. Additionally, *CaREM48*, *CaARF1*, *CaRAV4*, and *CaREM17* exhibited significant reductions in MG-BR-RR, with decreases of 4.68 times, 4.84 times, 6.76 times, and 12.55 times, respectively, during the RR period compared to the BR period.

### Subcellular location of the pepper *B3* genes

The Plant-mPLoc online tool served to explore the subcellular localization of *CaB3* proteins. The nucleus was predicted to contain the majority of *CaB3* proteins (Supplementary Table S1), where all *CaARF* proteins are found, consistent with previous research^40^. *CaRAV* proteins and *CaLAV* proteins are also located in the nucleus. 17 *CaREM* proteins were detected in both chloroplasts and the nucleus, whereas 47 *CaREM* proteins were only discovered in the nucleus. Chloroplasts held *CaREM30*, the cell membrane had *CaREM25*, the nucleus held *CaREM1*, and the cytoplasm held *CaREM1*. *CaREM2* was in the Golgi, and *CaREM11* and *CaREM57* were in the mitochondrion and the nucleus. These findings highlight the relative diversity in characteristics among members of the REM subfamily. The homologous gene *AtRAV1* of *CaRAV4* is involved in primary root development, leaf senescence, tree bud length, flowering, and possible general growth regulation. Therefore, the downregulated gene *CaRAV4* was selected to study its subcellular position, and a preliminary exploration was conducted on which cellular location *RAV4* may play a regulatory role in the development of pepper fruits^18,60^. We selected one of the down regulated genes, *CaRAV4*, to verify the prediction of subcellular localization. In order to determine the subcellular localization of *CaRAV4*, *CaRAV4*-GFP vector was constructed and transferred into Agrobacterium, and the bacterial solution was injected into the lower epidermis of well-grown *Nicotiana benthamiana* leaves by transient expression, and the subcellular localization was observed by confocal microscopy after 48 h of incubation protected from light. The results showed that the *CaRAV4*-GFP fusion protein appeared green fluorescence in the nucleus, and the GFP fluorescence emitted by the fusion protein overlapped with the nuclear localization signal mCherry (Fig. 10), indicating that *CaRAV4* localized in the nucleus. The location of *CaRAV4* in the nucleus of cells was further confirmed through the transient expression of *CaRAV4* in *Nicotiana benthamiana* leaves. So the evidence above suggests that most *CaB3* proteins primarily operate within the nucleus.

## Discussion

*B3* controls the growth of fruits, seeds, flowers, and the aging of leaves in reaction to various biological and non-biological pressures and the transmission of hormonal signals^20,21,33,46,61^. *B3* has been characterized genetically, and its associated functions have been studied in several species and have yielded valuable information^5,7–12^. We argue that this study is the first comprehensive exploration and analysis of the *B3* superfamily and its expression in pepper plants on a genome-wide scale. This study identified a total of 106 *CaB3s* using a comprehensive investigation of the complete genome. These proteins have been classified into four subfamilies by phylogenetic analysis (Fig. 1), aligning with the Arabidopsis *B3* protein grouping^1^, namely RAV, LAV, REM, and ARF subfamilies (Fig. 2). Pepper contains a lower amount of *B3* protein compared to mustard (118) and a higher amount compared to rice (91)^1^, despite having four subfamilies. In relation to the quantity of *ARF* and *REM* subfamily genes in pepper, we noticed a pattern comparable to that of other organisms—a predominantly inverse relationship between the number of *REM* and *ARF* genes present within a species^4^. Like Arabidopsis and rice, the *REM* subfamily contains numerous clusters of *B3* structural domain genes^1^. In peppers, it has been reported that there are nineteen *ARF* genes^40^. However, in this study, twenty-three *ARF* genes were identified, possibly due to the utilization of distinct parameters and additional selection criteria. The quantity of *REM* subgroups differs among different species, the role of the *REM* genes is seldom understood, and there could be notable duplications.

Exon–intron organization and motif analyses within gene families can further support and inform evolutionary relationships. The examination of various gene families from distinct species has revealed that closely related individuals typically possess conserved patterns and comparable arrangements of exons and introns within the same category. This finding supports the idea that these individuals have conserved functions and have evolved from a shared ancestor^62,63^. The current investigation involved the clustering and distribution of 106 *B3* proteins from pepper, which were categorized into four branches. It was observed that genes within the same branch exhibited a tendency to possess an equal number of introns and share similar conserved motifs, although there were a few exceptions (Fig. 6). When analyzing the intron–exon structure of the pepper *B3* genes, significant differences in the number and length of introns were found. This suggests that introns have either been lost or gained by these genes over the process of evolution. Pepper has been found to contain a total of eight *B3* genes without introns, possibly indicating the preference for intron removal to enhance the efficiency of replication, transcription, and processing, as suggested by previous research^64^. Additionally, variations in the number of exons in genes from the same subcategory have a substantial influence on the evolution of supergene families^65^. A motif, which is a brief and conserved sequence of amino acids, is regarded as the fundamental functional component necessary for the processing and folding of proteins^66^. The phylogenetic analysis's validity was confirmed by examination of the conserved pattern in the pepper’s *B3* gene since it revealed that genes belonging to the same branch of the phylogeny shared analogous motifs and had comparable motif organization. Furthermore, the presence of recurring motif patterns within individuals belonging to the identical subcategory of genes indicates duplication in both structure and function.

Studies on chromosomal localization in the past have indicated that genes belonging to the *REM* subfamily have a tendency to group together in the genome. Numerous species, including citrus, grapes, rice, tobacco, Arabidopsis, and others have shown this clustering phenomenon. In pepper, a comparable scenario was witnessed, wherein the most extensive gene cluster was situated on chromosome 1, close to the 300 Mb genomic area containing four *REM* genes (Fig. 3). Furthermore, it was discovered that numerous *CaB3* genes were grouped together in various sections of the remaining chromosomes. The large number of duplicate genes may have promoted the clustering of *REM* genes.

The expansion of gene families is mostly a result of tandem and segmental duplications, which play a vital role in the development of genes and gene families. In this process, segmental duplication plays a more significant function than tandem duplication^67^. In our current study, we discovered 11 instances of segmental duplication and four instances of tandem duplication in relation to the pepper *B3* gene. The results showed that the Ka/Ks ratios in the pepper *B3* superfamily were below 1. This shows that the evolution of all gene in the pepper *B3* superfamily has been impacted by purifying selection. In contrast to tandemly duplicated gene pairs (mean Ka/Ks = 0.54), segmentally duplicated gene pairs (mean Ka/Ks = 0.38) showed a variety of powerful purifying selects. Purification options are designed to maintain long-term stability by eliminating deleterious amino acid substitutions^68^. Therefore, it is essential to maintain and protect the *B3* gene family's important role in the development of pepper. In conclusion, our results are consistent with past studies in that the evolution of pepper *B3* genes predominantly by segmental duplication. Specifically, certain replicated genes were observed to be dispersed in sibling pairs within the phylogenetic tree, indicating their proximity to the shared progenitor. In addition, the high similarity of conserved motifs and exon–intron arrangements among duplicate genes suggests that they have functional redundancy in some biological processes. However, functional divergence between duplicate genes may have also occurred during long-term evolutionary processes.

The examination of collinearity can aid in the analysis of how the same gene family has evolved across various species. The collinearity of *B3* proteins among pepper, tomato, Arabidopsis, soybean, and rice was examined in this investigation (Fig. 5). The results showed that no colinearity was found between B3 in pepper and rice because although extensive homology and colinearity were identified in grasses and dicotyledons plants, there was much less homology and colinearity between the two populations due to longer evolutionary distances and more genomic rearrangements. Whereas pepper belongs to dicotyledons plants and rice belongs to grasses plants, no covariance of the B3 gene was found in pepper and rice, suggesting that it may be the long evolutionary distances and different genome rearrangements experienced by the two groups that have led to this phenomenon^69^. The findings also indicated that the quantity of direct similarity occurrences of *CaB3*-*SlB3* greatly surpassed that of *CaB3*-*OsB3*, implying that the separation of pepper and tomato, both belonging to the Solanaceae family, happened subsequent to the separation of the shared precursor of rice and dicotyledonous plants. Additionally, it suggested that pepper and tomato have a stronger genetic proximity to each other compared to soybean and Arabidopsis thaliana. The significant degree of collinear preservation between pepper and tomato implies that the *B3* transcription factor in pepper potentially possesses a comparable structure and function to the immediate homologous gene in tomato.

**Figure 8:**
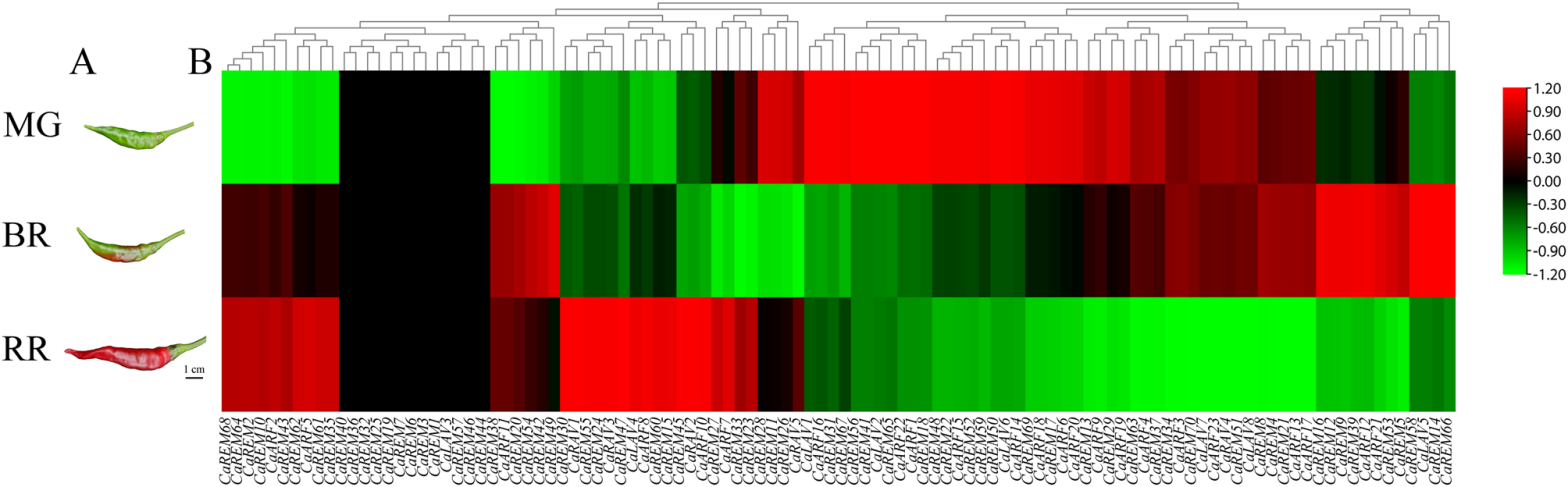
Heat map of 106 *CaB3* genes showing that they are highly expressed during pepper fruit ripening. (**A**). Pepper undergoes three distinct phases of fruit ripening: mature green fruit (MG, 40 DPA), breaker fruit (BR, which is partially red and occurs at 50 DPA), and red ripe fruit (RR, fully red and observed at 60 DPA). (**B**). Hierarchical clustering of *CaB3* genes in different fruit developmental periods. The scale bar indicates log_2_ normalized fragments per kilobase per million reads (FPKM) values. Red denotes high expression, while green denotes low expression, as shown by the color gradient.

The level to which genes are expressed at different developmental stages is closely related to the function of those genes. We learned more about the potential functions of these genes by examining how the *B3* genes in pepper expressed at different phases of fruit development. In the MG stage, *ARF*, *REM* genes, and a few *LAV* genes were highly expressed, except for *RAV* genes; specifically, the top three expression peaks were all *ARF* genes (*CaARF6*, *CaARF5*, *CaARF14*). During the BR stage, the majority of genes with high expression belonged to the *ARF* and *REM* subfamilies, with the exception of *CaLAV7*, *CaLAV4*, and *CaLAV8*. The three genes with the highest expression values were *CaARF5*, *CaREM53*, and *CaARF14*.During the RR phase, the *ARF* and *REM* subfamilies consisted of all highly expressed genes, excluding *CaLAV7*, *CaLAV4*, *CaRAV1*, and *CaLAV8*. Notably, the three highest expression peaks were attributed to *ARF* genes, namely *CaARF5*, *CaARF8*, and *CaARF14*.Based on this observation, it can be inferred that the elevated levels of auxin in the maturing pepper fruit stimulate the transcription of these *ARF* genes. Research observed a limited number of genes that were constantly expressed whereas a big number of genes were found to be selectively transcribed during particular developmental phases after analyzing transcriptome data during the three stages of pepper fruit development. *CaARF21*, *CaARF12*, and *CaARF17* exhibited an initial increase followed by a subsequent decrease throughout fruit development, reaching their highest point at the BR stage. This suggests that these three genes have the ability to control the entire process of fruit development. In tomato, the *SlARF8* gene represses the formation of fruit initiation^70^, whereas in our study *CaARF8* expression gradually increased during pepper fruit development and was higher at the red ripening (RR) stage, suggesting that *CaARF8* is also involved in the regulation of fruit initiation in pepper; down-regulation of *SlARF4* expression affects pectin synthesis and improves ripening fruit firmness^71^, however, *CaARF4* seems to be a positive regulator, and the expression of *CaARF4* gradually decreases with the decrease of fruit firmness in pepper; the repression of *SlARF7* transcription leads to the initiation of fruit set in tomato before pollination and fertilization^72^, and the expression of *CaARF7* decreases and then increases in the process of fruit development, which may play different roles at different periods of fruit development in pepper. *SlARF9* negatively regulates cell division in the early fruit development^73^, and it is possible that *SlARF9* negatively regulates cell division at the early stage of fruit development in pepper. *CaARF9* expression gradually decreases during the development of chili fruits, and cell division is not inhibited and the fruits are enlarged. In Arabidopsis, *ARF6* and *ARF8* coordinate the development of petals and reproductive organs during the transition from closed buds to mature fertile flowers, which contributes to efficient fertilization and subsequent fruit development^74^. *CaARF6* expression gradually decreased during pepper fruit development, while *CaARF8* expression gradually increased during pepper fruit development, which was different from the expression pattern in Arabidopsis. It is possible that there is no redundancy effect during pepper fruit development. Above of all, ARF is vital for regulating tomato fruit growth^43–47^. The majority of genes showed distinct reactions to the various stages of pepper fruit growth, based on the transcriptome data (Fig. 8). Meanwhile, some genes were highly expressed at all three developmental stages. *CaARF5* and *CaARF14* exhibited significant expression levels during the MG, BR, and RR phases, indicating their crucial involvement in the advancement of various developmental stages. *CaREM58*, *CaREM41*, and *CaREM56* were detected exclusively during a single developmental stage, indicating their specific involvement in a particular phase of fruit development. It was found that *CaREM36* and *CaREM32* in the tandem duplicate gene pair *CaREM35*/*CaREM36* and the segmental duplicate pair *CaREM29*/*CaREM32* were not expressed in all three fruit developmental periods, whereas *CaREM35* and *CaREM29* were expressed in all three fruit developmental periods, suggesting that the absence of expression of some *B3* genes may be due to gene duplication. The expression pattern of pepper *REM* genes is supported by earlier research on the expression and transcriptional characterization of *NtREM* as well as other *REM* genes^75,76^.

**Figure 9:**
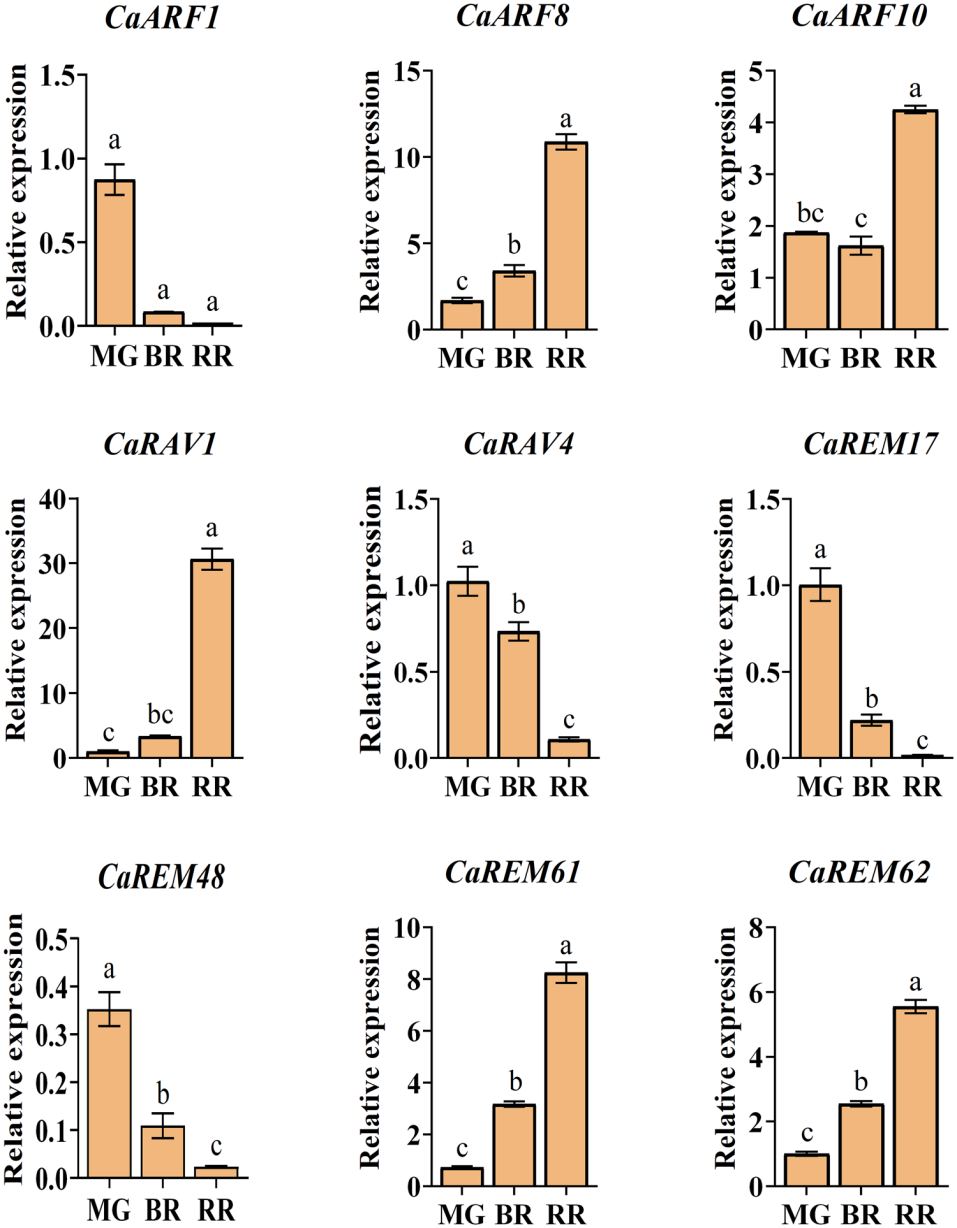
Relative expression of pepper *B3* genes during fruit development. Lowercase letters indicate significant differences. Distinct letters signify notable variations in levels of expression (*p* < 0.05, Student’s *t*-test).

**Figure 10:**
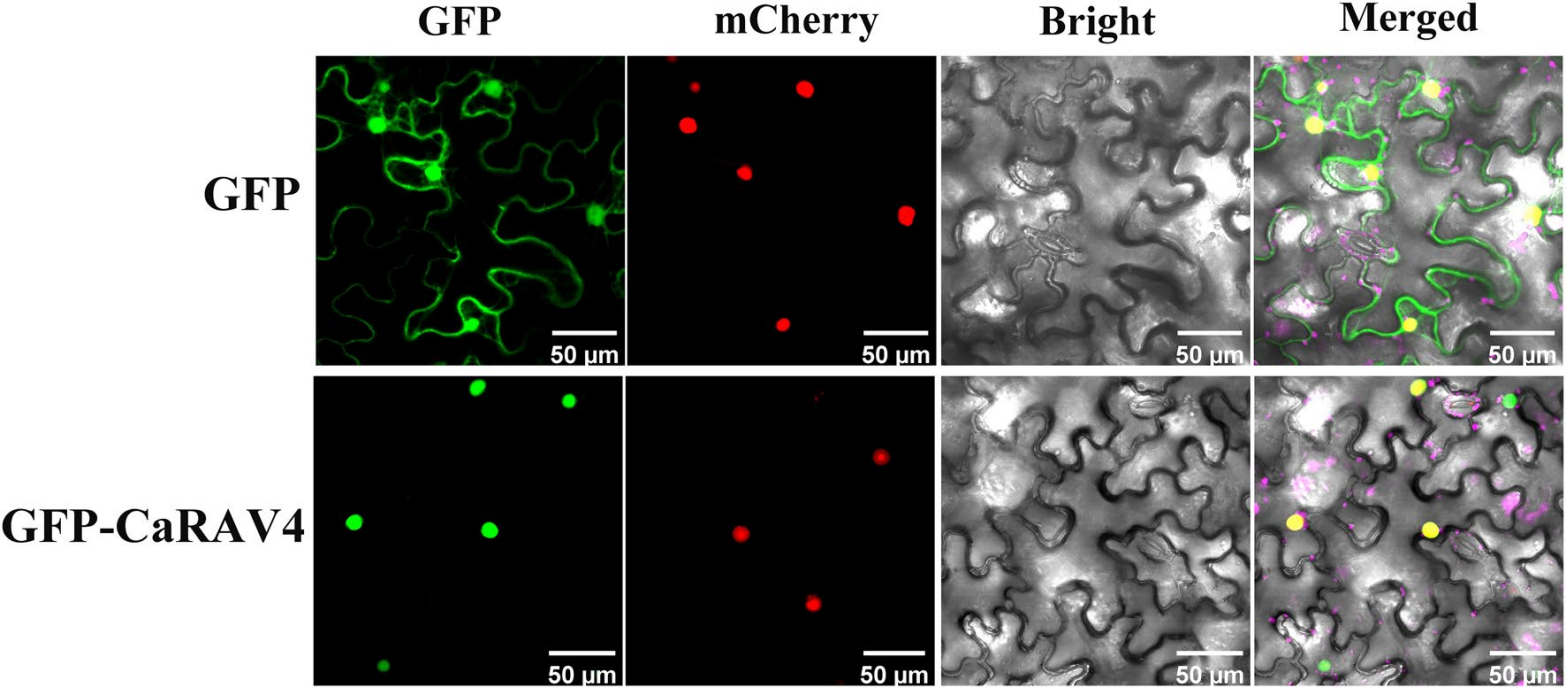
Subcellular location of the *CaRAV4*. The transgenic tobacco plants display images depicting the GFP signals of both formations. Confocal microscopy revealed the presence of green fluorescence. The protein mCherry is responsible for nuclear localization, while the protein GFP is responsible for green fluorescence. Visible light is used for bright field imaging, and the merged image combines the bright field with mCherry and GFP. Bar = 50 μm.

Furthermore, the findings were additionally confirmed through quantitative PCR (Fig. 9), suggesting that the *ARF* gene could potentially have a significant impact on fruit development, aligning with prior investigations^41,42^. During fruit development, the RR and MG phases exhibited a strong expression of the two *REM* genes, indicating their importance in fruit maturity. From the above, we can make a hypothesis that up-regulated genes are positively regulated and down-regulated genes are negatively regulated during the development of pepper fruits, and the inconsistency in the expression trend of some genes suggests that they play different roles at different times, and may be suppressed or facilitated by endogenous hormones within the stage that lead to this result. Future studies on the processes governing the growth and maturity of pepper fruit will benefit greatly from the insights provided by the *B3* gene. To learn more about the precise regulation mechanism of *B3* in the development of pepper fruit, a more comprehensive study is needed (Fig. 10).

## Conclusion

In this study, we conducted a systematic analysis of the *B3* gene family in Capsicum annuum. We identified the *B3* genes and analyzed their phylogenetic relationships, chromosomal distribution, gene structure, colinearity and its replication events, promoter elements, subcellular localization, and tissue-specific expression. The results of this study revealed the dynamic transcriptional patterns of *CaB3s* during fruit development, especially the ARFs genes may directly regulate the fruit development of pepper, suggesting that *CaB3s* may be involved in the formation and development of pepper fruits. The present study provides an important foundation and new insights for further research on the mechanism of *B3* genes regulating the development and maturation of pepper fruits, and provides a solid theoretical basis for the enhancement of the quality of peppers and the selection and breeding of their high-yield varieties. However, the specific molecular mechanisms of *B3* genes regulation in the development of pepper fruits should be studied more comprehensively and deeply in the future**.**

## Materials and methods

### Identification of *B3* genes in pepper

In order to accurately and comprehensively identify the *B3* superfamily in pepper, the genomic protein data of pepper was downloaded from the Ensemble Plants database (https://plants.ensembl.org Genomic version: Capsicum annuum cv.CM334 release 1.55) obtained^11^. We use Arabidopsis *B3* protein as the query sequence (https://www.arabidopsis.org/) Conducted BLAST search and then retrieved from Pfam database (http://pfam.xfam.org/) Download the HMM (Hidden Markov Model) map of the *B3* domain (PF02362), and use Hmmer 3.0 to re identify whether it contains the *B3* domain (with an e-value cutoff of 1e-5). After removing redundant sequences, all nonredundant proteins with conserved *B3* domains were assigned as members of the pepper *B3* family. Utilizing ExPASy (https://web.expasy.org/protparam/) Calculate the biochemical characteristics of the *B3* gene family in chili peppers. The subcellular localization of each CaB3 protein was predicted using the Plant-mPLoc online tool (http://www.csbio.sjtu.edu.cn/bioinf/plant-multi/)^77^.

### Phylogenetic analysis of the pepper *B3* genes

The *B3* protein from pepper was compared to the downloaded amino acid sequences of Arabidopsis *B3* using ClustalW with the default parameters^78^. The maximum likelihood (ML) technique was used to build an important phylogenetic tree with 1000 bootstrap replicates using IQ-TREE (multicore version 2.2.0)^79^.

### Chromosomal distribution and conserved motif analyses of *B3* in pepper

Data regarding the placement of the *B3* gene in pepper chromosomes was obtained from the Genome General Characterization File. The chromosomal locations of Pepper *B3* were determined using the internet resource MG2C (http://mg2c.iask.in/mg2c_v2.1/). MEME (http://meme-suite.org/) was used to examine the conserved motifs of the pepper *B3* gene, with a maximum of 15 conserved motifs set^80^. Visualization of the genetic structure and conservative sequence of the pepper *B3* family was done using TBtools^81^.

### Cis-acting element, collinearity, and duplication analysis of pepper *B3* genes

We were able to obtain a 2000 bp section of the pepper *B3* gene sequence before the start codon using TBtools. We next used the PlantCare website (http://bioinformatics.psb.ugent.be/webtools/plantcare/html/) to submit this extracted sequence for analysis of its cis-acting elements^82^. To assess the collinearity of *B3* genes within and between species and to map collinearity, we utilized MCScanX^83^ and Circos^84^. Furthermore, the Ka/Ks values of the *B3* gene pairs were calculated using the (natural gradient descent, NG) method by TBtools in order to understand the selection pressure on the *B3* gene in pepper during the process of evolution.

### Plant materials

The tested pepper (*C. annuum*) is a self-selected ’chili pepper inbred line gd-75–3-10 by the research group. It was cultivated in a light-culture room with alternating periods of 16 h of light and 8 h of darkness at a temperature of 22–25 °C. At the time of fruiting, fruit samples were gathered from MG fruits (40 DPA), BR fruits (partially red fruits, 50 DPA), and RR fruits (fully red fruits, 60 DPA). Three sets of biological duplicates were enclosed in aluminum foil, rapidly frozen in liquid nitrogen, and preserved at a temperature of − 80 °C. Afterward, the specimens were preserved at a temperature of − 80 °C. The samples were stored simultaneously.

### Transcriptome sequencing to analyze *CaB3* gene expression levels

For transcriptome analysis of the mentioned samples of MG fruit (40 DPA), BR fruit (partially red fruits, 50 DPA), and RR fruit (completely red fruits, 60 DPA) of pepper, three biological replicates were collected. Novogene (Beijing, China)^85^ performed the extraction of total RNA, creation and testing of libraries for quality, as well as the sequencing, analysis, and annotation of the transcriptome. The R package was used to generate heatmaps and to standardize expression levels using FPKM values. The "full" clustering approach was used to conduct a hierarchical cluster analysis. FPKM (fragments per kilobase per million mapped reads) was used to represent these transcripts.

### qRT-PCR analysis

*B3* genes with significantly different expression levels were screened among the three maturation stages. These genes were chosen from the RNA-seq data due to their significant variations in transcript abundance across the stages, with a relatively high multiplicity (|log2FC|≥ 1, *P* ≤ 0.05). To ascertain the expression patterns, nine genes were chosen for qRT-PCR analysis. For qRT-PCR analysis of the mentioned samples of MG fruit (40 DPA), BR fruit (partially red fruits, 50 DPA), and RR fruit (completely red fruits, 60 DPA) of pepper, three biological replicates were collected, the RNA-prep Pure Total RNA Extraction Kit for Polysaccharide-Polyphenol Plants (Tiangen, Beijing, China) was utilized for extraction. Sangyo Bioengineering Corporation (Shanghai, China) designed and synthesized the Total RNA primers, as listed in Supplementary Table S7. The RNA that was extracted underwent reverse transcription to cDNA with the FastKing gDNA Dispelling RT SuperMix (Tiangen, Beijing, China). Fluorescence quantitative PCR was conducted utilizing an ABIVi-iA7 instrument (Hangzhou, China). *CaUBI-3* was reported to have the most stable expression level in pepper under abiotic stress and different tissue types; therefore, this gene can be recommended as the best reference gene for qRT-PCR analysis in pepper^86^. Gene expression levels were determined by employing the 2^−ΔΔCt^ technique^87^. The data were examined for statistical significance and visualized using GraphPad Prism 8.0. The significance level of 0.05 was used to conduct multivariate comparisons through one-way ANOVA and Duncan's multiple range test.

### Subcellular localization analysis

To confirm the anticipated outcomes, *Nicotiana benthamiana* plants were chosen for transient expression using *CaRAV4*.To generate a vector of *CaRAV4*-GFP under the control of the P35S promoter, the coding sequence of *CaRAV4* was combined with the N-terminus of GFP in pEGOEP35S-H-GFP. Agrobacterium strain GV3101 carrying the *CaRAV4*-GFP vector was mixed in equal proportions with the Agrobacterium strain GV3101 with the mCherry Nucleus marker before infiltration, and the infiltrated leaves were directly observed with an FV1000 laser confocal microscope after 48 h of infiltration. The emission filters for GFP and mCherry were set at 488 nm and 561 nm, correspondingly.

### Supplementary Information

Supplementary Figure S1.

Supplementary Table S1.

Supplementary Table S2.

Supplementary Table S3.

Supplementary Table S4.

Supplementary Table S5.

Supplementary Table S6.

Supplementary Table S7.

## Data Availability

The self-tested RNA-Seq data of pepper fruits at different fruit stages in this study can be obtained at the Short Read Archive (SRA) of NCBI with BioProject PRJNA1017123.

## Material availability

The pepper material used for the experiment is a self-selected and purified strain by our research group, and has not yet been registered as a variety. We have the right to collect plants, conduct experimental and field research on peppers, and comply with relevant institutional, national, and international standards and legislation.
