# Supplementary figures and images for: Genome-wide identification of the B3 transcription factor family in pepper (Capsicum annuum) and expression patterns during fruit ripening
Source: Sci Rep. 2024 Jan 26;14:2226. doi: 10.1038/s41598-023-51080-6 (PMC10817905; doi:10.1038/s41598-023-51080-6)

Figure S1: Graph of Motif Results

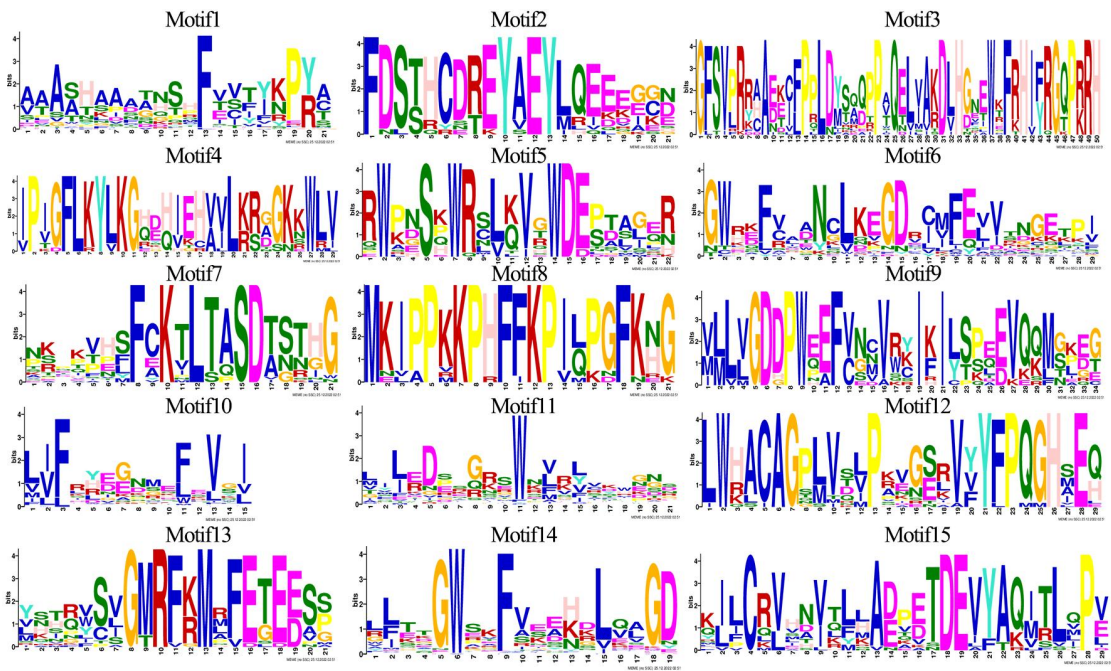

Supplement: Supplementary file 1 — Supplementary Figure S1. [file 41598_2023_51080_MOESM1_ESM.pdf]
